# Quality of life among patients with autoimmune hepatitis in remission: A comparative study

**DOI:** 10.1097/MD.0000000000022764

**Published:** 2020-10-23

**Authors:** Atsushi Takahashi, Masanori Abe, Tetsuya Yasunaka, Teruko Arinaga-Hino, Kazumichi Abe, Akinobu Takaki, Takuji Torimura, Mikio Zeniya, Kaname Yoshizawea, Jong-Hon Kang, Yoshiyuki Suzuki, Nobuhiro Nakamoto, Ayano Inui, Atsushi Tanaka, Hajime Takikawa, Hiromasa Ohira

**Affiliations:** a Department of Gastroenterology, Fukushima Medical University School of Medicine, Hikarigaoka, Fukushima; b Department of Gastroenterology and Metabology, Ehime University Graduate School of Medicine, Shitsukawa, To-on, Ehime; c Department of Gastroenterology and Hepatology, Okayama University Graduate School of Medicine, Dentistry, and Pharmaceutical Sciences, 2-5-1 Shikata-cho, Kita-ku, Okayama-city; d Department of Medicine, Kurume University School of Medicine, 67 Asahi-machi, Kurume-shi, Fukuoka; e Sanno Medical Center, International University of Health and Welfare, 8-10-16 Akasaka, Minato-ku, Tokyo; f Department of Gastroenterology, National Hospital Organization, Shinshu Ueda Medical Center, 1-27-21 Midorigaoka, Ueda-City, Nagano; g Center for Gastroenterology, Teine Keijinkai Hospital, 1-12 Maeda, Teine-ku, Sapprro; h Department of Hepatology, Toranomon Hospital, Toranomon 2-2-2, Minato-ku; i Department of Internal Medicine, Keio University School of Medicine, 35 Shinanomachi, Shinjuku-ku, Tokyo; j Department of Pediatric Hepatology and Gastroenterology, Saiseikai Yokohamashi Tobu Hospital, 3-6-1 Shimosueyoshi, Tsurumi-ku, Yokohama-City, Kanagawa; k Department of Medicine, Teikyo University School of Medicine, 2-11-1, Kaga, Itabashi-ku; l Faculty of Medical Technology, Teikyo University, 2-11-1, Kaga, Itabashi-ku, Tokyo, Japan.

**Keywords:** autoimmune hepatitis, chronic hepatitis C, disease duration, primary biliary cholangitis, quality of life, remission

## Abstract

Health-related quality of life (HRQOL) is lower in individuals with autoimmune hepatitis (AIH) than in the general population. However, previous evaluations of HRQOL for AIH have included a broad range of disease activities. The aim of this study was to clarify HRQOL among patients with AIH in remission.

We assessed HRQOL in patients with AIH in remission, patients with chronic hepatitis C (CHC) with eradicated hepatitis C virus (HCV) and patients with primary biliary cholangitis (PBC) using the Japanese version of the Chronic Liver Disease Questionnaire (CLDQ).

Participants comprised 62 patients with AIH in remission, 39 patients with CHC with eradicated HCV and 66 patients with PBC. Median ages of patients were 63, 69, and 64 years, respectively. Overall score (5.6 vs 5.9, *P* = .02) and fatigue (5.2 vs 5.6, *P* = .01) and worry (5.6 vs 6.0, *P* = .01) domain scores of the CLDQ were significantly lower in patients with AIH in remission than in CHC with eradicated HCV, and similar to scores except for the systemic symptoms domain in patients with PBC. Disease duration was associated with lower scores on systemic symptoms and activity domains of the CLDQ in patients with AIH in remission.

Patients with AIH in remission show impaired HRQOL associated with disease duration.

## 1 Introduction

Health-related quality of life (HRQOL) is an essential treatment target in patients with chronic liver disease, due to the associations with prognosis.^[1,2]^ The HRQOL of patients with autoimmune hepatitis (AIH) is lower than that of the general population.^[3–7]^

German single-center study reported depression and anxiety has been dominant symptoms in patients with AIH.^[4]^ Moreover, Polish single-center study described depression in patients with AIH was not associated with clinical and biochemical features of AIH.^[7]^ On the other hands, we have previously reported that impaired HRQOL in AIH is impacted by patient background characteristics such as cirrhosis, comorbidities, and high-dose prednisolone.^[5]^

Disease control is important for both the prognosis and HRQOL of patients with chronic liver disease. Direct antiviral agents (DAAs) against hepatitis C virus (HCV) can not only eradicate the virus, but also improve HRQOL in patients with chronic hepatitis C (CHC).^[8,9]^ In AIH, the immunosuppressants can reduce disease activity and allow patients to achieve remission, but little is known regarding HRQOL among patients in remission.

Corticosteroids are established immunosuppressants in patients with AIH, but show various side effects including obesity, hyperglycemia, hyperlipidemia, and osteoporosis. In Japan, AIH patients usually continue low-dose corticosteroids after remission to avoid relapse. We therefore considered that AIH patients may show poor HRQOL despite being in remission. This study analyzed HRQOL among AIH patients in remission in comparison with other controlled liver diseases such as patients with CHC with eradication of HCV and patients with primary biliary cholangitis (PBC), and investigated associated factors.

## 2 Methods

### 2.1 Study population

We conducted this study among members of the Autoimmune Hepatitis Study Group, a subgroup of the Intractable Hepato-biliary Disease Study Group in Japan. Patients with AIH, CHC with eradicated HCV and PBC were recruited consecutively at each participating hospital. Diagnosis AIH and PBC were made based on established criteria, respectively.^[10,11]^ In addition to patients with CHC with eradicated HCV, only AIH patients with remission and controlled PBC patients were included in this study. Remission in patients with AIH was defined as normal alanine transferase (ALT) and immunoglobulin G (IgG) at the time of answering the CLDQ. However, patients were included as “in remission” if the attending physician judged remission despite abnormal levels of ALT and/or IgG. Relapse of AIH was defined as serum ALT level ≥60 U/L after corticosteroid treatment and serum ALT normalization (≤30 U/L). Controlled PBC was also defined as normal alkaline phosphatase (ALP) and γ-glutamyl transpeptidase (γGTP) at the time of completing the CLDQ. Patients were included in this study if the attending physician judged the individual as well-controlled despite abnormal levels of ALP and/or γGTP. Among 39 patients with CHC with eradicated HCV, 30 patients (76.9%) and 9 patients (23.1%) were treated by DAAs and interferon-based regimen, respectively. Patients with presence or past history of liver cirrhosis, malignant disease, or mental disorder were excluded. All subjects were enrolled between January 2019 and March 2020 and written informed consent was obtained from all subjects. The study protocol conformed to the ethical guidelines of the 1975 Declaration of Helsinki and was approved by the ethics committee at Fukushima Medical University (no. 29187).

### 2.2 Questionnaire

We asked each participant to complete the self-reported Japanese version of the Chronic Liver Disease Questionnaire (CLDQ). The original version of the CLDQ includes 29 items divided into 6 subdomains: abdominal symptoms (3 items), fatigue (5 items), systemic symptoms (5 items), activity (3 items), emotional function (8 items), and worry (5 items).^[12]^ The Japanese version of the CLDQ has already been published and statistically validated.^[13]^

### 2.3 Physical measurements and data collection

To analyze the effects of physical factors on HRQOL, we performed physical measurements. Handgrip strength was measured using a hand dynamometer (TL110; TOEI LIGHT CO., LTD., Saitama, Japan). Appendicular skeletal muscle index (ASMI) was measured by bio-impedance analysis using an Inbody770 body composition analyzer (Inbody, Seoul, Korea). Bone mineral density (BMD) was measured at the lumbar spine (L2-L4) using dual-energy X-ray absorptiometry (GE Healthcare, Madison, WI, USA). Clinical and demographic data were collected from patient records at each clinic.

### 2.4 Statistical analyses

Results are presented as median and interquartile range. Two groups were compared using the Mann–Whitney *U* test for continuous variables. For CLDQ scores, analysis of covariance was performed to determine any significant differences between groups, after adjusting for age and sex. Correlations between variables were measured using Spearmans rank correlation to examine associations between patient background and HRQOL among patients with AIH. If values were missing, statistical analysis was performed using available data. Statistical analysis was performed using SPSS for Windows version 25.0 (SPSS, Chicago, IL, USA), and values of *P* < .05 were considered indicative of significant differences.

## 3 Results

### 3.1 Clinical and demographic data

A total of 62 patients with AIH, 39 patients with CHC, and 66 patients with PBC were enrolled. Median ages of patients were 63, 69, and 64 years, respectively (Table 1). Median age was significantly lower in patients with AIH than in patients with CHC. The proportion of women was lower in patients with AIH (83.4%) than in patients with PBC (92.4%, *P* = .005). Median duration of disease was 68 months (interquartile range, 32–163 months) and 94 months (interquartile range, 28–185 months) in patients with AIH and PBC, respectively. Median duration from end of treatment for HCV was 36 months (interquartile range, 28–50 months). Among 64 patients with AIH, 35 patients (54.7%) experienced relapse (mean number of relapses, 1.7). Albumin level was significantly lower in patients with AIH than in patients with CHC. Levels of ALT, ALP, and γGTP were significantly lower in patients with AIH than in patients with PBC. Compared to patients with PBC, frequency of comorbid diseases was higher in patients with AIH. Frequency of medication for osteoporosis was significantly higher in patients with AIH than in patients with CHC or PBC. BMD was higher in patients with AIH than in patients with CHC. No differences in ASMI were apparent between groups.

**Table 1:**
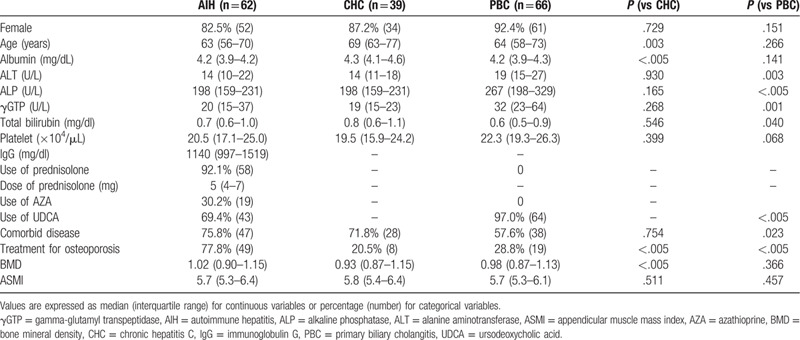
Patient characteristics.

### 3.2 CLDQ domain scores

**Figure 1:**
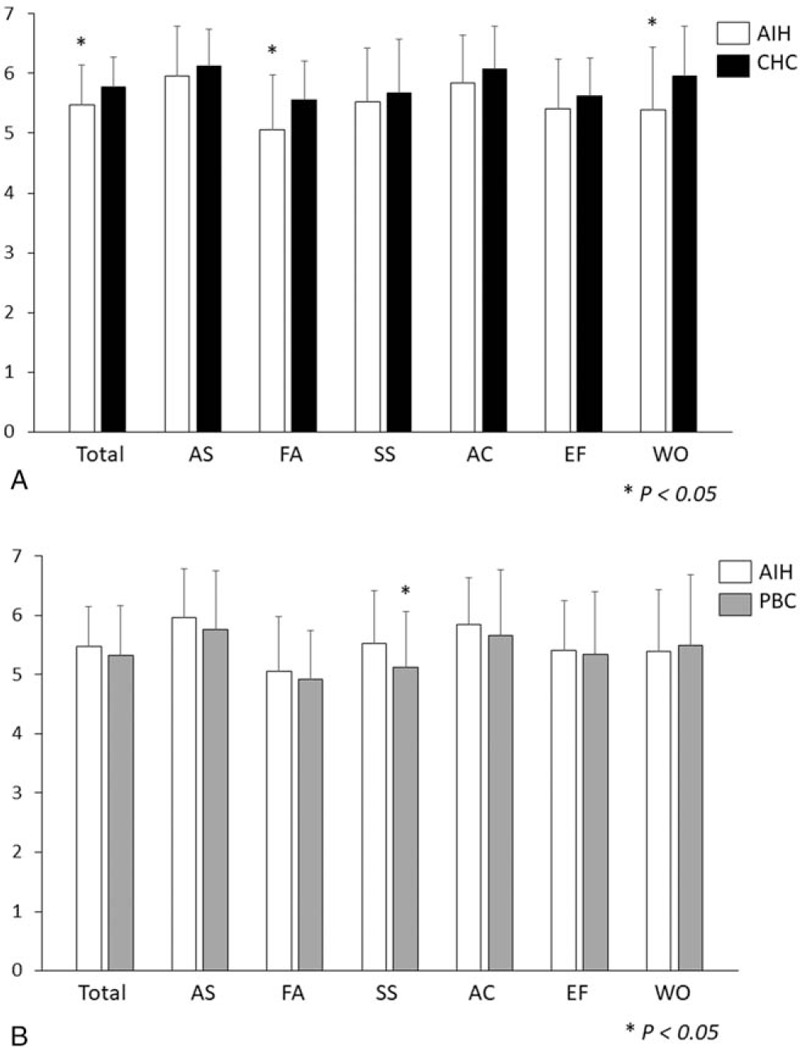
Comparisons of Chronic Liver Disease Questionnaire (CLDQ) scores. (A) Comparison of CLDQ scores between patients with autoimmune hepatitis (AIH) in remission and chronic hepatitis C (CHC) with eradicated hepatitis C virus (HCV). (B) Comparison of CLDQ scores between patients with AIH in remission and primary biliary cholangitis (PBC). Analysis of covariance was performed to identify significant differences between 2 groups, after adjusting for age and sex. AC = activity, AS = abdominal symptoms, EO = emotional function, FA = fatigue, SS = systemic symptoms, WO = worry.

Figure 1A shows the comparison of CLDQ scores between patients with AIH and CHC. Overall scores and subdomain scores for fatigue and worry were significantly lower in patients with AIH than in patients with CHC. Figure 1B shows the comparison of CLDQ scores between patients with AIH and PBC. Only the subdomain score for systemic symptoms was lower in patients with PBC than in patients with AIH.

### 3.3 Associations between backgrounds and HRQOL among patients with AIH

Regarding the associations between backgrounds and HRQOL among patients with AIH, only disease duration correlated negatively with domain scores for systemic symptoms (rs = −0.30, *P* = .016) and activity (rs = −0.31, *P* = .013). Although no significant relationship was identified, handgrip strength tended to correlate positively with activity domain scores (rs = 0.25, *P* = .141) (Table 2).

**Table 2:**
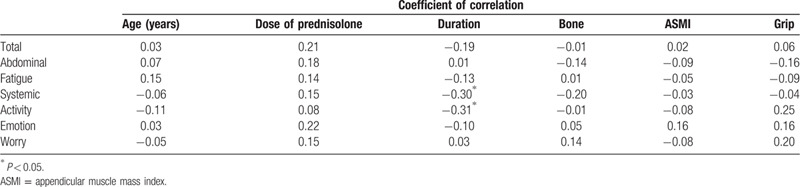
Associations between backgrounds of patients with AIH and CLDQ score.

## 4 Discussion

The present study evaluated HRQOL among patients with AIH in remission using the CLDQ. We found that HRQOL was more impaired for patients with AIH in remission than for patients with CHC with eradicated HCV, and was relatively comparable to that of patients with PBC. In addition, HRQOL of patients with AIH in remission was significantly associated with disease duration. To the best of our knowledge, this study is the first to compare HRQOL between patients with AIH in remission and CHC with eradicated HCV or PBC.

Lower HRQOL in patients with CHC may be explained by many factors, such as liver disease stage, comorbidities, extrahepatic lesions, and disease perception.^[14–17]^ DAAs for HCV can improve the HRQOL for patients with CHC.^[8,9]^ However, HRQOL for patients with CHC has been reported to remain lower than that of the general population even after viral clearance.^[18]^ Although we did not directly compare HRQOL between AIH and healthy individuals in this study, the above report implies that HRQOL among patients with AIH in remission remains lower than that of the general population. In fact, we retrospectively confirmed the lower HRQOL of patients with AIH in remission compared to healthy controls from our previous survey in 2018 (supplementary figure).

Impaired HRQOL has been reported for patients with PBC in many studies.^[19–21]^ We also showed that HRQOL was impaired and lower serum albumin level was independently associated with fatigue scores from the short-form 36 questionnaire among Japanese PBC patients.^[22]^ Although patients with AIH and PBC both showed impaired HRQOL, few reports have provided direct comparisons. Only one study compared HRQOL between patients with AIH and patients with cholestatic disease, including PBC and primary sclerosing cholangitis.^[3]^ That study reported similar HRQOL, in agreement with the present study.

Impaired HRQOL has been reported patients with AIH, especially focusing on depression and anxiety.^[4,7]^ We have also reported that impaired HRQOL among patients with AIH was associated with factors such as disease progression, comorbid diseases, and prednisolone treatment.^[5]^ We therefore focused on and analyzed patients with AIH in remission in this study, to minimize the above background effects. This yielded different results from the previous report. Disease duration was the only factor associated with impaired HRQOL in the present study. On the other hand, previous studies including our own have shown that disease duration was not associated with HRQOL among patients with AIH. This difference may be due to several reasons. First, patients with AIH in past studies were younger than those in the present study. Age correlates significantly with worsening HRQL in patients with chronic liver disease.^[23]^ Second, this study analyzed only AIH patients in remission and without cirrhosis or active hepatitis. Differences in disease activity can have differing effects on HRQOL. Third, methods for evaluating HRQOL differed, except in comparison with our previous study. Although the direct effect of disease duration on HRQOL is unclear, we found a positive association with disease duration and times of relapse (rs = 0.36, *P* < .05). We therefore believe that the experience of relapse during a long clinical course is one reason for the association with disease duration and impaired HRQOL. The association between prolonged clinical remission and better HRQOL in patients with systemic erythematosus seems to indirectly reinforce our result.^[24]^

Sarcopenia, low muscle mass and low muscle strength result in a deteriorated clinical course of liver disease.^[25,26]^ In addition, low muscle mass is associated with impaired HRQOL among patients with liver disease. Although we could not identify an association between ASMI and HRQOL, hand grip tended to be positively associated with the activity domain. Effects of hand grip on QOL have been reported in digestive diseases, including liver disease in Japan.^[26]^ The effects of skeletal mass or handgrip on HRQOL may thus be worth reanalyzing in a larger sample size.

Osteoporosis is one critical side effect of corticosteroids and is common in liver disease.^[27]^ Although osteoporosis is largely asymptomatic, osteoporotic fractures result in the deterioration of HRQOL.^[28]^ On the other hand, the BMD of AIH patients in remission was significantly higher than that of CHC in the present study, because of the higher frequency of patients receiving treatment for osteoporosis. This suggests proper or preventive treatment for osteoporosis was being performed more frequently in patients with AIH in remission. In fact, no AIH patients in the present study experienced bone fracture.

The present study showed several limitations. First, the sample size was small, and we may therefore have overlooked factors significantly associated with HRQOL among AIH patients in remission. Validation and reanalysis using unified evaluation methods need larger samples in the future. Second, patients with AIH in remission may be different in Japan from those in other countries. For example, most patients with AIH in remission were controlled by low-dose corticosteroids in Japan. On the other hand, patients in other countries may keep in remission under other drugs such as azathioprine and mycophenolate mofetil. Multicenter study in England reported AIH patients with corticosteroid treatment reduced QOL regardless of dose of corticosteroid or disease activity.^[6]^ Therefore, the effect of corticosteroid on HRQOL may not ignore in present study. Third, we used CLDQ to evaluate HRQOL, which is an established method for chronic liver diseases including PBC.^[10,29]^ However, detailed evaluation of HRQOL in patients with PBC may have been unsatisfactory compared to that from the PBC-40, a PBC-specific measure of symptoms and HRQOL.^[19]^ Despite these limitations, one strength of the present survey was the new findings based on comparison with unique participants in addition to analyses using ASMI, handgrip and BMD.

## 5 Conclusions

The HRQOL of AIH patients was lower despite them being in remission. Disease duration was associated with impaired HRQOL, especially for systemic symptom and activity domains. Physicians should pay attention to the HRQOL of AIH patients with longer disease duration, even if they are in remission.

## Author contributions

All authors contributed to the study conception and design. Data collection and analysis were performed by Atsushi Takahashi, Masanori Abe, Tetsuya Yasunaka, Teruko Arinaga-Hino and Kazumichi Abe. The first draft of manuscript was written by Atsushi Takahashi and Hiromasa Ohira. All Authors read and approved the final manuscript.

**Conceptualization:** Atsushi Takahashi, Masanori Abe, Tetsuya Yasunaka, Teruko Arinaga-Hino, Kazumichi Abe, Akinobu Takaki, Takuji Torimura, Mikio Zeniya, Kaname Yoshizawea, Jong-Hon Kang, Yoshiyuki Suzuki, Nobuhiro Nakamoto, Ayano Inui, Atsushi Tanaka, Hajime Takikawa, Hiromasa Ohira.

**Data curation:** Atsushi Takahashi, Masanori Abe, Tetsuya Yasunaka, Teruko Arinaga-Hino, Kazumichi Abe.

**Formal analysis:** Atsushi Takahashi.

**Funding acquisition:** Hajime Takikawa.

**Investigation:** Atsushi Takahashi, Masanori Abe, Tetsuya Yasunaka, Teruko Arinaga-Hino.

**Project administration:** Atsushi Takahashi, Masanori Abe, Tetsuya Yasunaka, Teruko Arinaga-Hino, Kazumichi Abe, Akinobu Takaki, Takuji Torimura, Mikio Zeniya, Kaname Yoshizawea, Jong-Hon Kang, Yoshiyuki Suzuki, Nobuhiro Nakamoto, Ayano Inui, Atsushi Tanaka, Hajime Takikawa, Hiromasa Ohira.

**Supervision:** Hiromasa Ohira.

**Writing – original draft:** Atsushi Takahashi.

**Writing – review & editing:** Atsushi Takahashi, Hiromasa Ohira.

## Supplementary Material

Supplemental Digital Content
