# Supplementary material for: Quality of life among patients with autoimmune hepatitis in remission: A comparative study
Source: Medicine (Baltimore). 2020 Oct 23;99(43):e22764. doi: 10.1097/MD.0000000000022764 (PMC7581115; doi:10.1097/MD.0000000000022764)

Supplementary figure

The comparison of Chronic Liver Disease Questionnaire (CLDQ) scores between patients with autoimmune hepatitis (AIH) in remission and healthy controls in a 2018 survey. Analysis of covariance was performed to identify significant differences between groups, after adjusting for age and sex. AS, abdominal symptoms; FA, fatigue; SS, systemic symptoms; AC, activity; EO, emotional function; WO, worry.


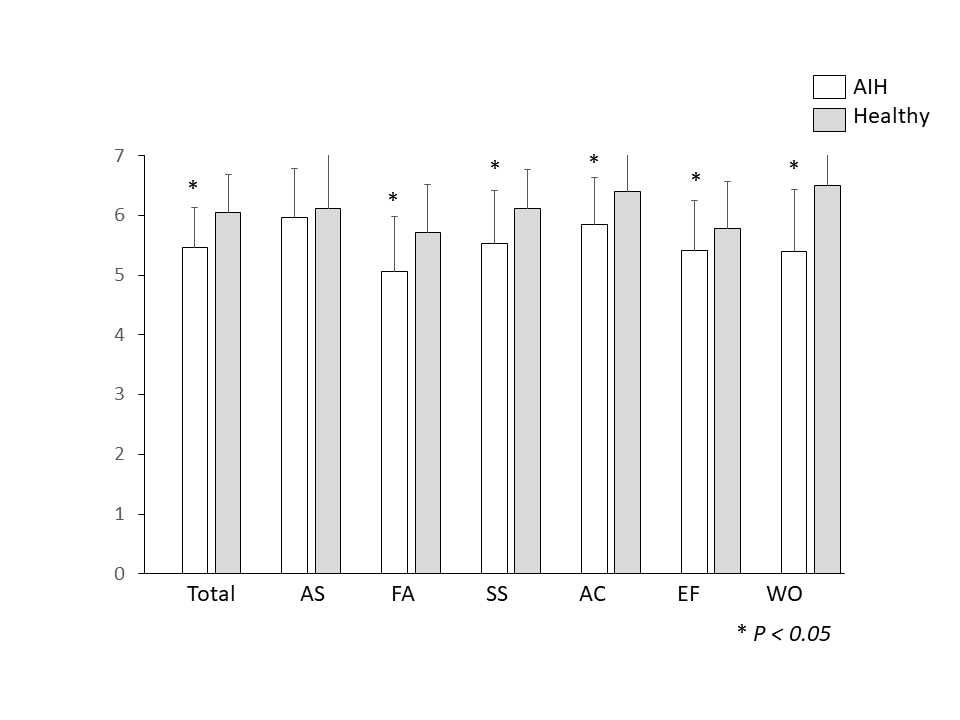

Supplement: Supplemental Digital Content [file medi-99-e22764-s001.docx]
